# HM-Swin: Hierarchical Multiscale Swin Transformer Network with Attention Fusion for Heart Sound Classification

**DOI:** 10.3390/bioengineering13101188

**Published:** 2026-10-12

**Authors:** Kalpeshkumar Ranipa, Wei-Ping Zhu, M. N. S. Swamy

**Affiliations:** Department of Electrical and Computer Engineering, Concordia University, Montreal, QC H3G 1M8, Canada; weiping@ece.concordia.ca (W.-P.Z.); swamy@ece.concordia.ca (M.N.S.S.)

**Keywords:** deep learning, attention mechanism, transformer, heart sound classification

## Abstract

Heart sound classification is crucial in the early detection and diagnosis of cardiovascular diseases, aiming at effectively capturing the complex patterns of heart sound signals. Deep learning models have widespread applications in heart sound classification, but the existing methods solely rely on either early, late or intermediate fusion, which often fail to fully integrate the diverse and multiscale features required for robust classification. To overcome these limitations, this study proposes a novel hierarchical multiscale swin transformer network with attention fusion (HM-Swin), for heart sound classification. The new model incorporates a swin transformer backbone to extract multiscale features spanning from low-level to high-level representations. These features are further refined through a dynamic fusion block, wherein the features extracted by each swin block are enhanced using dedicated channel-wise and spatial attention mechanisms. This approach effectively captures interdependencies, resulting in a robust and comprehensive feature representation. Extensive experiments conducted on publicly available benchmark datasets, namely, PhysioNet2016 and PhysioNet2022, achieve classification accuracies of 98.32% and 90.56%, respectively, demonstrating that HM-Swin achieves higher performance than existing state-of-the-art methods. These results confirm the efficiency and scalability of our model, showing its potential as a solution for automated heart-sound screening and classification.

## 1. Introduction

According to the World Health Organization (WHO), cardiovascular disease (CVD) remains a prevalent condition that poses a significant threat to global health—a leading cause of mortality worldwide, accounting for approximately 17.9 million deaths annually or 32% of all global fatalities. Importantly, more than 75% of these deaths occur in low- and middle-income countries, with a significant portion resulting from myocardial infarctions and cerebrovascular accidents [1]. Particularly in the United States, CVD was responsible for nearly one in five deaths in 2022, with over 700,000 individuals succumbing to heart-related conditions [2]. Historically, auscultation of heart sounds has been a prevalent method for detecting abnormalities in heart valves as an initial diagnostic step. The method of analyzing phonocardiogram (PCG) signals is widely utilized to monitor physiological changes in myocardial valves for the purpose of identifying abnormalities or diseases. Generally, a skilled physician examines heart sounds using a stethoscope, identifies irregularities, and provides an initial diagnosis. However, the process of auscultation through human auditory perception is inherently challenging and demands considerable expertise. The reason for this is that the human ear may overlook certain acoustic elements, as higher-frequency sounds can obscure those of lower frequencies. Therefore, there is a critical need to explore efficient and rapid diagnostic methodologies for cardiovascular disease. In [3], the authors investigate traditional machine-learning-based methods for predicting heart disease. However, the performance of these methods is highly susceptible to noise in the recorded signals.

Some researchers utilized both PCG and electrocardiogram (ECG) signals to improve heart disease classification performance [4,5], and segmentation task [6]. These studies demonstrate that using both signals results in better classification outcomes compared to using only PCG signals [7]. Despite these, synchronizing the ECG and PCG signals in a clinical setting presents difficulties. Furthermore, obtaining ECGs requires the patient to lie supine with electrodes attached to various body regions, potentially compromising patient comfort and privacy. In contrast, PCG is a non-invasive and more patient-friendly technique, requiring only the placement of an electronic stethoscope on the chest. Consequently, PCG serves as a practical and effective approach for early CVDs diagnosis.

In recent years, deep learning frameworks have attained significant traction across numerous tasks. A variety of neural architectures, including convolutional neural networks (CNNs), recurrent neural networks (RNNs), attention mechanisms, and transformers, have been employed to heart sound classification tasks. Among these, CNNs are proficient in efficiently extracting localized features by utilizing parameter sharing through convolutional operations, and they demonstrate superior generalization capabilities with more rapid convergence. The authors in [8] utilize mel-frequency cepstral coefficients (MFCCs) features derived from a short-time Fourier transform (STFT), which proficiently encapsulate both the temporal and spectral characteristics of the heart-sound signals. These features are subsequently fed into a pretrained VGG16 network for further classification. In [9], the authors utilized a ResNet framework for partitioned iterated function systems (PIFS) features. However, these standalone CNN architectures are often inadequate. Therefore, numerous researchers have embedded attention mechanisms into their models to enhance the performance of classification tasks by focusing on the most relevant features. In [10], to enhance classification accuracy, the author employed bispectral analysis for feature extraction, which was then fed into the ResNet model followed by an attention block. Similarly, in [11], the authors extracted time-domain features and then employed a 1D convolutional architecture along with a channel attention mechanism. The authors in [12] designed a cascaded multi-module architecture, where each module contains convolutional layers and a channel attention mechanism to perform deep feature extraction. The resulting feature maps are then fed into fully connected layers followed by a softmax layer for classification. The researchers utilized a CNN-MHA framework in [13], firstly applying a residual CNN, followed by multi-head attention (MHA) that leverages diverse features, such as time-frequency domain characteristics, along with peak interval and boundary detection information from fundamental heart sounds S1 and S2. It can be seen that these attention strategies, along with CNN, enhance classification performance by concentrating on the most relevant features. However, these methods often exhibit limitations in capturing long-range dependencies within the signal, making them inadequate for effectively processing complex signals such as heart sounds. Consequently, several researchers have opted to employ a hybrid framework wherein features extracted by CNNs are subsequently processed by RNNs to effectively capture dependencies. In [14], continuous wavelet transform (CWT) is applied to obtain a detailed time-frequency representation of the input signals. A CNN–LSTM model is then utilized, where the CNN extracted spatial features, followed by a long short-term memory (LSTM) network to capture temporal dependencies. In [15], the authors proposed a CNN and bidirectional gated recurrent units (GRUs)-based architecture with MFCCs as the input. In contrast to cascade CNN networks, several researchers have employed parallel architectures to capture features at various resolutions. In [16], a two-branch network is proposed, where one branch uses a CNN and the other employs a multilayer perceptron (MLP) network. The authors in [17] propose a dual-branch CNN framework to extract complementary features, where one branch processes high-order spectral features, while other utilizes short-time Fourier transform (STFT) features. Moreover, the authors in [18] and [19] utilize a three-branch CNN framework tailored to leverage the benefits of diverse features, with each branch processing distinct domain-specific inputs. In [20], a multi-branch architecture is designed wherein each branch incorporates a CNN–LSTM network, enabling the joint modeling of spatial and temporal features. Nevertheless, these CNN-based techniques can lead to the loss of spatial information during convolution and pooling processes, potentially causing suboptimal performance in tasks that demand the capture of global dependencies.

To mitigate the limitations of CNN-based methods, which primarily emphasize local connections, transformer [21] has been explored in computer vision due to its ability to capture long-range dependencies. Vision transformer (ViT) [22] demonstrates superior performance capabilities and greater architectural versatility. ViTs excel in comprehensive sequence modeling, making them particularly advantageous for classification task [23,24]. In [25], the authors employed a ViT to extract long-range dependencies and intricate patterns in heart sound signals from mel-spectrogram features. Various refinements to ViT have been introduced to create more efficient network architectures, facilitating effective deployment without the necessity for extensive pre-training on large datasets. Consequently, numerous researchers have adopted hybrid models to boost performance over employing a single model. In [26], the authors utilized a convolutional ViT that takes continuous wavelet transform (CWT) spectrogram as input, initially employing inception-v3 as the convolutional layers, followed by a ViT. Similarly, the authors in [27], employed a CNN-transformer hybrid network with raw signal input, where local feature extraction is performed by CNNs and global sequence modeling is done by transformers. In [28], the authors employed a residual 1D CNN-transformer architecture, integrating CNN layers and residual connections in the encoder block, which takes MFCCs and their delta features as input. In [29], the authors introduced a filter-based transformer model comprising three stages: first, a CNN to extract time-domain features; second, a learnable filtering layer to capture frequency-domain periodicities; and third, a transformer encoder to model global dependencies. Furthermore, in [30], a pre-trained CNN–Transformer framework is adopted, where a segmentation step is applied prior to feeding the input into the model. The authors in [31] adapted the cascade wav2vec framework, which takes raw heart sound signals as input. However, the above-mentioned cascaded hybrid methods are configured sequentially, so each layer’s performance relies on the output generated by its predecessor. In other words, ViT’s capacity to model global features is significantly shaped by the local features derived from the CNN. This nature of interdependence can constrain the architecture’s ability to grasp detailed context at each phase, diminishing its efficiency compared to parallel architectures that enable concurrent feature extraction across multiple levels. The authors in [32] devised an architecture where ViT and RNN operate in parallel to process spatial and temporal data, with these features combined at the feature level. In [33], the authors employ a parallel structure of convolutional modules and transformer-based attention modules to capture the local and global features of heart murmurs effectively. In [34], the researchers designed a dual-branch network employing late fusion with a max operation, where one branch processes spectrogram features and the other manages time-domain features. Each branch integrates CNN layers followed by a transformer encoder block to extract diverse feature representations. The authors in [35] developed DTF-STCANet, where swin transformer and ConvNeXt branches process spectrogram and CWT representations in parallel, with the fused features classified using a weighted KNN classifier. In [36], the authors utilized a three-branch parallel network consisting of two CNN branches and a multi-head transformer encoder branch, with feature-level fusion before feeding into the classifier. Even though these hybrid architectures have demonstrated encouraging results with feature fusion happening at early, intermediate and late stages, they lack the capability to capture multiscale dependencies proficiently, constraining their potential to represent hierarchical feature representation from the signal information.

Despite the fact that ViT enhances classification accuracy, it typically demands substantial datasets for fine-tuning due to its data-intensive nature [37]. On the other hand, swin transformer [38] follows a hierarchical structure that limits self-attention computations to localized windows, reducing computational costs and providing scalability across various classification tasks. More specifically, it is important to highlight that the swin transformer, with its shifted window mechanism and hierarchical configuration, adeptly captures both local and global patterns in structured data and provides a more streamlined approach to capture multiscale features. Heart sounds encompass information across a range of frequency bands and temporal scales, making multiscale feature extraction an essential aspect of improving classification performance. In this paper, we aim to integrate the multiscale features with the swin transformer model. Furthermore, we propose a multiscale attention fusion (MAF) module that synthesizes multiscale features into the classification vector, enabling the model to prioritize the most pertinent features for classification.

Our key contributions are summarized below:

1. We develop HM-Swin, a hierarchical framework for heart sound classification that effectively leverages the swin transformer backbone with multiscale feature representations. These features are extracted at different network depths: shallow layers capture fine-grained local characteristics, while deeper layers emphasize global contextual information. This hierarchical multiscale representation ensures a comprehensive and discriminative feature representation.
2. We propose a novel MAF module that integrates multiscale feature maps from each stage of the swin transformer, leveraging both spatial and channel attention mechanisms. This design enhances the model’s capacity to understand complex patterns and relationships within the data.
3. Our proposed architecture is evaluated on two publically available datasets through extensive experimentation to validate its effectiveness. The results reveal that the HM-Swin not only outperforms existing state-of-the-art techniques but also significantly reduces computational complexity by lowering the parameter count.

The organization of this paper is as follows. Section 2 presents the proposed HM-Swin architecture and elaborates on its key components, beginning with the feature space transformation, followed by the deep feature extractor and the multiscale attention fusion module. Section 3 presents the experimental setup and a comprehensive simulation study with comparison to baseline models and state-of-the-art methods. Finally, Section 4 concludes the paper.

## 2. Proposed HM-Swin Architecture

**Figure 1:**
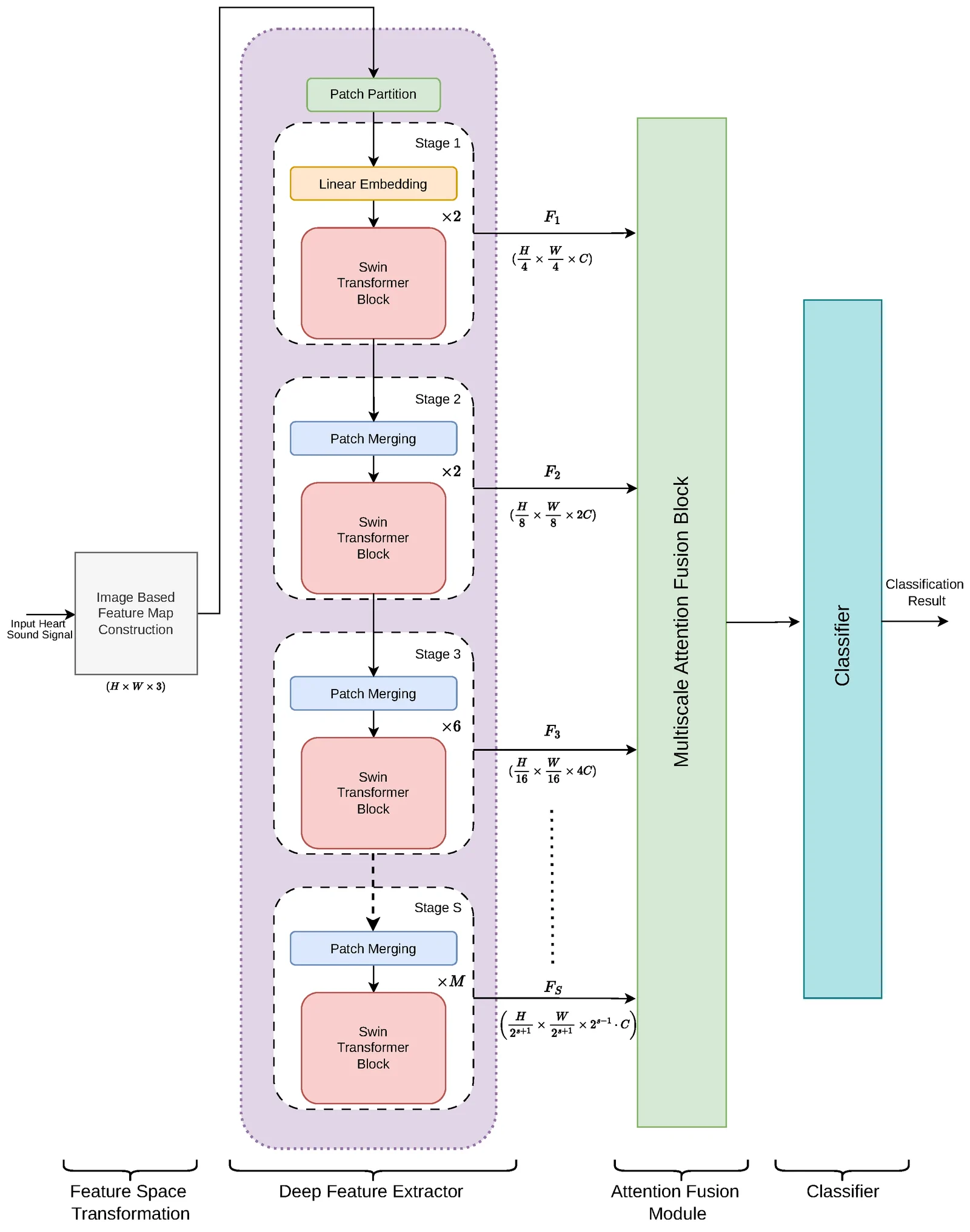
The comprehensive architectural overview of the proposed HM-Swin framework.

Figure 1 depicts the overall architecture of the proposed HM-Swin framework, which is primarily composed of a deep feature extractor with a swin transformer-based backbone network, a multiscale attention fusion block, and a classifier. Initially, input heart sound signals are transformed into an image-based representation using feature space transformation. Next, the transformed features are fed into a deep feature extractor, where the swin transformer-based backbone effectively captures multiscale representations by leveraging its hierarchical structure to extract diverse features across multiple layers. The MAF module is structured as a feature pyramid, which contains multiple adaptive attention modules (AAM). The multiscale features generated by the deep feature extractor are fed into the corresponding stages of the AAM module. Finally, the fused feature maps are concatenated and passed through the MLP layers to produce the prediction result. Detailed descriptions of the proposed model and its key components are provided in the subsequent subsections.

### 2.1. Feature Space Transformation

In this work, MFCCs are computed and arranged into an image-based feature representation, which is then used as input to the transformer-based network for feature extraction. To generate the feature map, MFCCs along with their delta and double-delta coefficients, are employed to form a three-dimensional representation, which effectively captures the salient characteristics of complex heart sound signals. MFCCs are calculated as per following sequential steps. The process begins with dividing the heart sound signal into frames using a Hamming window. Next, the discrete-time Fourier transform (DTFT) is applied to each frame, and the squared magnitude spectrum is computed. This is followed by calculating the filterbank energies by summing the energy contributions within each mel-scaled triangular filterbank. The log-mel filterbank energies are then obtained by applying a logarithmic transformation to the filterbank energies. Finally, the discrete cosine transform (DCT) is performed on the log-mel filterbank energies, resulting in the MFCCs, which effectively represent the spectral features of the signal. In this study, 64 MFCCs are calculated per frame. To construct a 2D image-based feature representation, 64 consecutive frames are considered, resulting in a 64 × 64 matrix. In order to enhance the temporal feature representation, delta and double-delta features are computed for each frame and arranged as additional channels. Consequently, the final input to the proposed HM-Swin network is a three-channel representation, where the first channel comprises MFCC features, the second includes delta features, and the third contains double-delta features. Herein, we used a window size of 1024 samples with no overlap, and one feature representation is generated per PCG recording.

### 2.2. Deep Feature Extractor

The transformer faces several challenges in the domain of computer vision (CV) due to fundamental differences between CV and natural language processing (NLP). A major challenge in this domain is the rapid variation in the scale of signal components. Additionally, the signals are often contaminated with noise during the acquisition process in clinical environments. To address these challenges, the swin transformer has been introduced [38]. It constructs hierarchical feature maps and computes attention only within local windows, rather than globally. This approach effectively reduces computational complexity. To maintain connectivity across windows, the swin transformer uses a shifted window strategy, where the window partitions are shifted after computing attention within each window. This design not only minimizes the computational burden but also allows the model to handle various scales with greater adaptability. The HM-Swin proposed in this article can more effectively capture less signal variations through a hierarchical window for feature enhancement. The following sections present a comprehensive description of patch embedding and patch merging, followed by an overview of the self-attention mechanism and swin transformer block.

#### 2.2.1. Patch Embedding and Patch-Merging

As detailed in Section 2.1, following the feature space transformation, the resulting image-based feature representation has dimensions of 64×64×3. In the proposed network, this input image-based feature-map data is converted to 1D data and down-sampled using patch embedding and patch merging, respectively. Each of these patches acts as a ‘token’, with its feature representation being the concatenation of the raw pixel values of the feature map. In our configuration, a patch size of 4 × 4 is employed, resulting in a feature dimension of 4 × 4 × 3 = 48 per patch. A linear embedding layer is subsequently applied to these features, mapping them into a custom-defined dimensional space denoted as C. A series of swin transformer blocks is then applied to these patch tokens. As shown in Figure 1, the patch embedding is performed only in stage 1 and serves as the first layer of the proposed HM-Swin network. In this layer, the H and W dimensions of the input feature maps are merged together, thus transforming the feature map from two dimensions to one dimension. These 1D feature maps are further passed through a swin transformer block. To generate a hierarchical structure, the token count is progressively reduced through patch merging layers as the network deepens. In stages 2 to S, a patch merging layer is employed before each swin transformer block. This layer down samples the extracted features to decrease their resolution, thereby producing hierarchical and multiscale features similar to those in a standard CNN. The resulting down-sampled feature map is half the size of the original feature map input. Specifically, in the initial three stages, as features are processed through the swin transformer blocks, the feature map resolution reduces, and the channel dimension expands following each patch merging operation. The patch merging layer aggregates features from adjacent 2 × 2 blocks and then applies a linear transformation along the channel axis, reducing the number of patches by a factor of four, halving the spatial resolution, and doubling the channel dimension. For the generalized case, at each stage *s*, the spatial resolution and channel dimensionality of the output feature map are given by $\frac{H}{2^{s+1}}\times \frac{W}{2^{s+1}}\times 2^{s-1}\cdot C$, respectively. In our configuration, we employ a four-stage hierarchical structure to fully exploit multi-level feature representations across varying resolutions. Beyond stage four, additional downsampling is not feasible since the spatial resolution of the feature maps becomes insufficient to support further patch merging operations.

At stage 1, which consists of a linear embedding layer and a swin transformer block, produces the feature map output with the dimension of $\frac{H}{4}\times \frac{W}{4}$. Subsequently, swin transformer blocks are employed for feature transformation, maintaining the resolution at $\frac{H}{8}\times \frac{W}{8}$. This phase of patch merging and transformation is labeled as ‘stage 2’. The same process is repeated twice, referred to as ‘stage 3’ and ‘stage 4’, yielding output resolutions of $\frac{H}{16}\times \frac{W}{16}$ and $\frac{H}{32}\times \frac{W}{32}$, respectively. These stages collectively construct a hierarchical representation. In summary, the output resolutions for the four stages are $\frac{H}{4}\times \frac{W}{4}$, $\frac{H}{8}\times \frac{W}{8}$, $\frac{H}{16}\times \frac{W}{16}$ and $\frac{H}{32}\times \frac{W}{32}$, with respective channel dimensions of *C*, 2C, 4C, and 8C.

#### 2.2.2. Fundamentals of Attention Mechanism

The attention mechanism serves as the central component of the transformer, which dynamically assigns importance to various parts of the input. It enables this input to interact and focus on the most relevant elements. Also, it identifies which components should receive greater focus. This mechanism highlights the significance of each input (e.g., token) in its current context, calculated as an attention score. The final output represents the aggregation of these interactions, each weighted by its respective attention score. More precisely, It uses three vectors—query, key, and value, the mechanism matches a query with a set of key-value pairs to generate the output. The attention mechanism is categorized into scaled dot-product attention and multi-head attention.

The queries and keys share the same dimensionality, $d_{q}=d_{k}$, while the values have a dimensionality of $d_{v}$. To compute the attention, the dot product between the query and each key is calculated, and this result is divided by a scaling factor, $\sqrt{d_{k}}$, and then passed through a softmax function. The resulting attention weights are subsequently applied to the values via a dot product operation. In practice, the queries, keys, and values are formulated as matrices *Q*, *K*, and *V*, respectively, and the attention score is computed as follows:

(1) $$\mathrm{Attention}\left(Q,K,V\right)=\mathrm{Softmax}\left(\frac{QK^{T}}{\sqrt{d_{k}}}\right)V$$

Multi-head attention enhances the model’s ability to represent diverse features and provides more stability during training when compared to single-head attention. It enables the model to focus on information from different subspaces and positions simultaneously. To achieve this, the queries, keys, and values are projected multiple times, corresponding to the number of attention heads (*h*). Each head processes the attention mechanism in parallel, and the results are then concatenated and reprojected to produce the final output. The operation of multi-head self-attention (MSA) can be formulated as,

(2) $$\mathrm{MSA}\left(Q,K,V\right)=\mathrm{Concat}\left({\mathrm{head}}_{1},\ldots ,{\mathrm{head}}_{h}\right)W^{O}$$

where each ${\mathrm{head}}_{i}$ is determined by:

(3) $${\mathrm{head}}_{i}=\mathrm{Attention}\left(QW_{i}^{Q},KW_{i}^{K},VW_{i}^{V}\right)$$

where, $W_{i}^{Q}\in R^{d_{\mathrm{model}}\times d_{k}},\;W_{i}^{K}\in R^{d_{\mathrm{model}}\times d_{k}},\;W_{i}^{V}\in R^{d_{\mathrm{model}}\times d_{v}},\;W^{O}\in R^{hd_{v}\times d_{\mathrm{model}}}$ are the projection matrices for the *i*-th head, and $W^{O}$ is the output projection matrix. The $d_{\mathrm{model}}$ refers to the dimension of the queries, keys, and values.

#### 2.2.3. Swin Transformer Blocks

**Figure 2:**
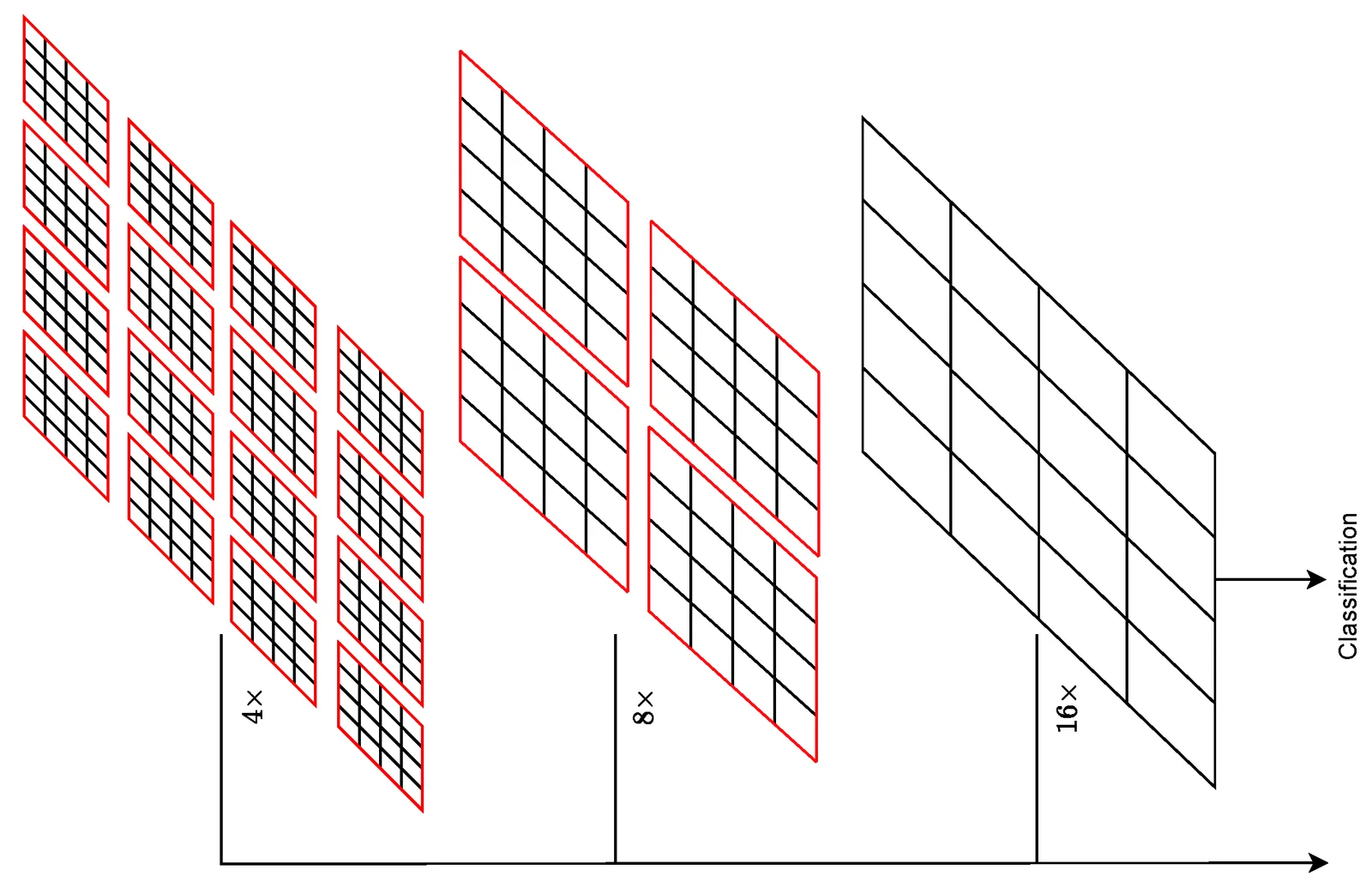
Feature map representation in the hierarchical feature mapping technique.

The swin transformer is an advanced transformer-based model that serves as a versatile backbone for various applications. As the network deepens, the earlier ViT maintains the same downsampling operations to generate feature maps, which remain continuous without further division. In contrast, the swin transformer employs a hierarchical mapping strategy, as demonstrated in Figure 2, which closely resembles the configuration of CNNs. At the initial stage, the input images are divided into non-overlapping patches through a patch partitioning module. These patches are progressively merged within the deeper layers of the transformer. This approach reduces computational complexity from quadratic to linear by applying self-attention within non-overlapping windows. Nevertheless, this partitioning may reduce connectivity between individual windows. To tackle this issue, the swin transformer incorporates a shifted window technique enabling the exchange of information across windows from the preceding layer, which results in a significant expansion of the model’s receptive field. This strategy represents a key distinction from traditional transformer architectures.

**Figure 3:**
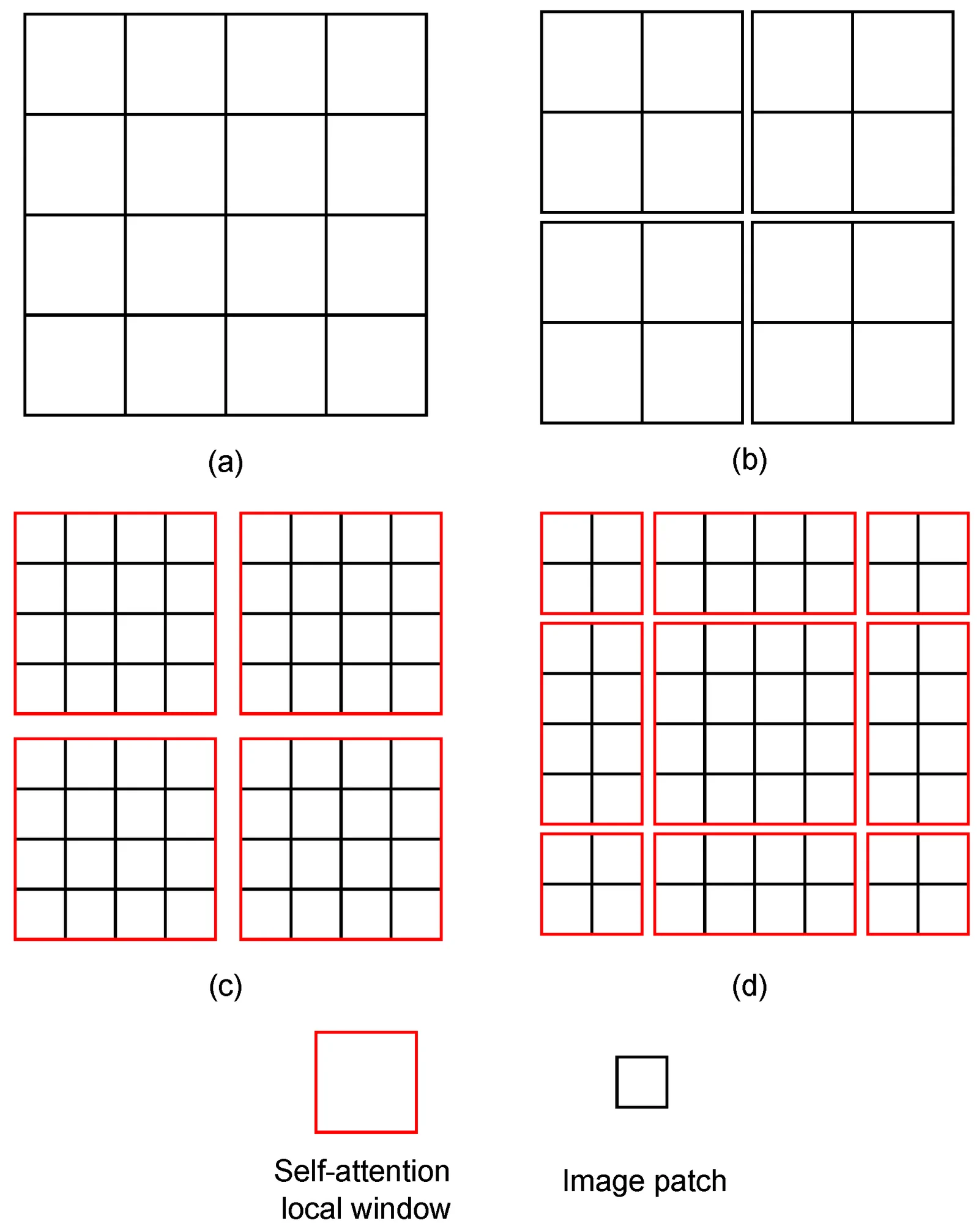
The figure illustrates: (**a**,**b**) the window multi-head self-attention (W-MSA) mechanism, and (**c**,**d**) the shifted window multi-head self-attention (SW-MSA) mechanism.

Unlike the conventional MSA mechanism, our proposed method employs window-based multi-head self-attention (W-MSA) and shifted window-based multi-head self-attention (SW-MSA) for applying spatial attention to heart sound image-based feature representations [38]. The shifted window technique inherent in W-MSA and SW-MSA facilitates the extraction of feature representations across multiple spatial resolutions while minimizing the computational complexity [21]. The W-MSA and SW-MSA are applied successively across these modules. As depicted in Figure 3a,b, it computes the self-attention for each pixel relative to others within a localized window. It divides the feature map into multiple windows, where self-attention is executed independently for each window. Within the W-MSA mechanism, self-attention is limited to individual windows, prohibiting cross-window interaction among these windows. To address this, the SW-MSA module is utilized. As shown in Figure 3c,d, this module shifts the window positions compared to the W-MSA. The shifted windows enable interaction with multiple neighboring windows, significantly expanding the receptive field.

The computational complexity for MSA and W-MSA is expressed in Equation (4) and Equation (5), respectively:

(4) $$\Omega_{\mathrm{MSA}}=4hwC^{2}+2{\left(hw\right)}^{2}C$$

(5) $$\Omega_{W\text{-}\mathrm{MSA}}=4hwC^{2}+2M^{2}hwC$$

where *C* denotes the feature dimension. When *M* is fixed (typically set to 7), the computational complexity of W-MSA scales linearly with the product of *h* and *w*. In the proposed HM-Swin configuration, for feature maps whose spatial resolution is smaller than the nominal window size, the effective window size is limited to the feature-map resolution.

**Figure 4:**
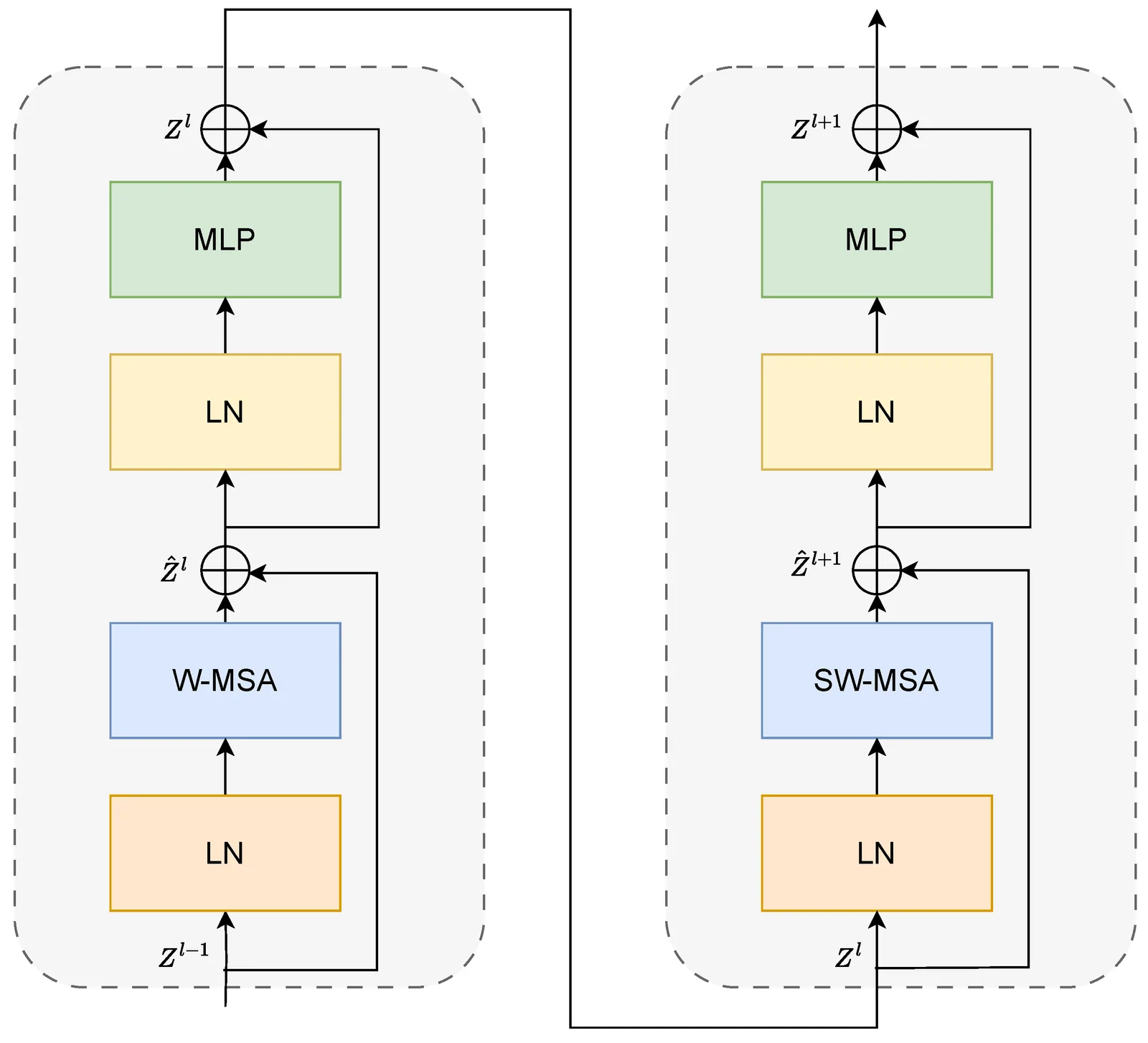
Two successive swin transformer blocks: W-MSA on the left and SW-MSA on the right.

As depicted in Figure 1, the proposed HM-Swin transformer architecture contains four stages of the swin transformer blocks. The configuration of the swin transformer block is illustrated in Figure 4; it includes two consecutive swin transformer blocks as a basic unit. The first block uses a multi-head attention module with regular window configurations, while the second block features a shifted window multi-head attention module. The MLP is composed of two layers with a GELU activation function between them, and both blocks incorporate layer normalization. Swin transformer block can be mathematically defined as follows:

(6) $${\hat{z}}^{l}=W\text{-}\mathrm{MSA}\left(\mathrm{LN}\left(z^{l-1}\right)\right)+z^{l-1}$$

(7) $$z^{l}=\mathrm{MLP}\left(\mathrm{LN}\left({\hat{z}}^{l}\right)\right)+{\hat{z}}^{l}$$

(8) $${\hat{z}}^{l+1}=\mathrm{SW}\text{-}\mathrm{MSA}\left(\mathrm{LN}\left(z^{l}\right)\right)+z^{l}$$

(9) $$z^{l+1}=\mathrm{MLP}\left(\mathrm{LN}\left({\hat{z}}^{l+1}\right)\right)+{\hat{z}}^{l+1}$$

Here, ${\hat{z}}^{l}$ and $z^{l}$ denote the outputs of the (S)W-MSA module and the MLP module at the *l*th block, respectively, and the self-attention is computed as follows.

(10) $$\mathrm{Attention}\left(Q,K,V\right)=\mathrm{Softmax}\left(\frac{QK^{T}}{\sqrt{d}}+B\right)V$$

where *Q*, *K*, and $V\in R^{\left(M^{2}\times d\right)}$ represent the query, key, and value matrices. $M^{2}$ and *d* indicate the number of image patches and the dimensionality of the query or key within a window. The value of *B* are calculated from the deviation matrix $\hat{B}\in R^{\left(2M-1)\times (2M-1\right)}$. The traditional transformer block computes self-attention by associating each patch with every other patch, resulting in a quadratic escalation in the network’s computation relative to the image’s resolution.

### 2.3. Multiscale Attention Fusion Module

As explained in Section 2.2.1, the deep feature extractor generates hierarchical three-dimensional feature representations $F_{s}$, s=1,2,3,4, with dimensions structured as H×W×C (representing height, width, and channels) across 4 different stages. More specifically, these feature maps can be denoted as: $F_{1}\in R^{\frac{H}{4}\times \frac{W}{4}\times C},\;F_{2}\in R^{\frac{H}{8}\times \frac{W}{8}\times 2C},$$\;F_{3}\in R^{\frac{H}{16}\times \frac{W}{16}\times 4C},\;F_{4}\in R^{\frac{H}{32}\times \frac{W}{32}\times 8C}$. The swin transformer’s output already preserves the spatial layout and channel depth. Although it is feasible to generate multiscale feature maps directly using the swin-based backbone network, a standalone classifier may not be sufficient to capture the underlying information embedded within these features. Therefore, the MAF module is introduced to integrate information from multiple layers, specifically the output of the swin transformer-backbone network, resulting in multiscale feature maps that provide a more comprehensive representation of context. The MAF module combines features from multiple layers of the swin transformer-backbone network, enriching the contextual information conveyed in the feature maps. As a result, inspired by the convolutional block attention module (CBAM) [39], we implemented the adaptive attention module (AAM) to strengthen the discriminative properties of these multiscale feature maps. The AAM leverages both channel and spatial attention mechanisms, improving the feature maps’ ability to capture and represent information.

**Figure 5:**
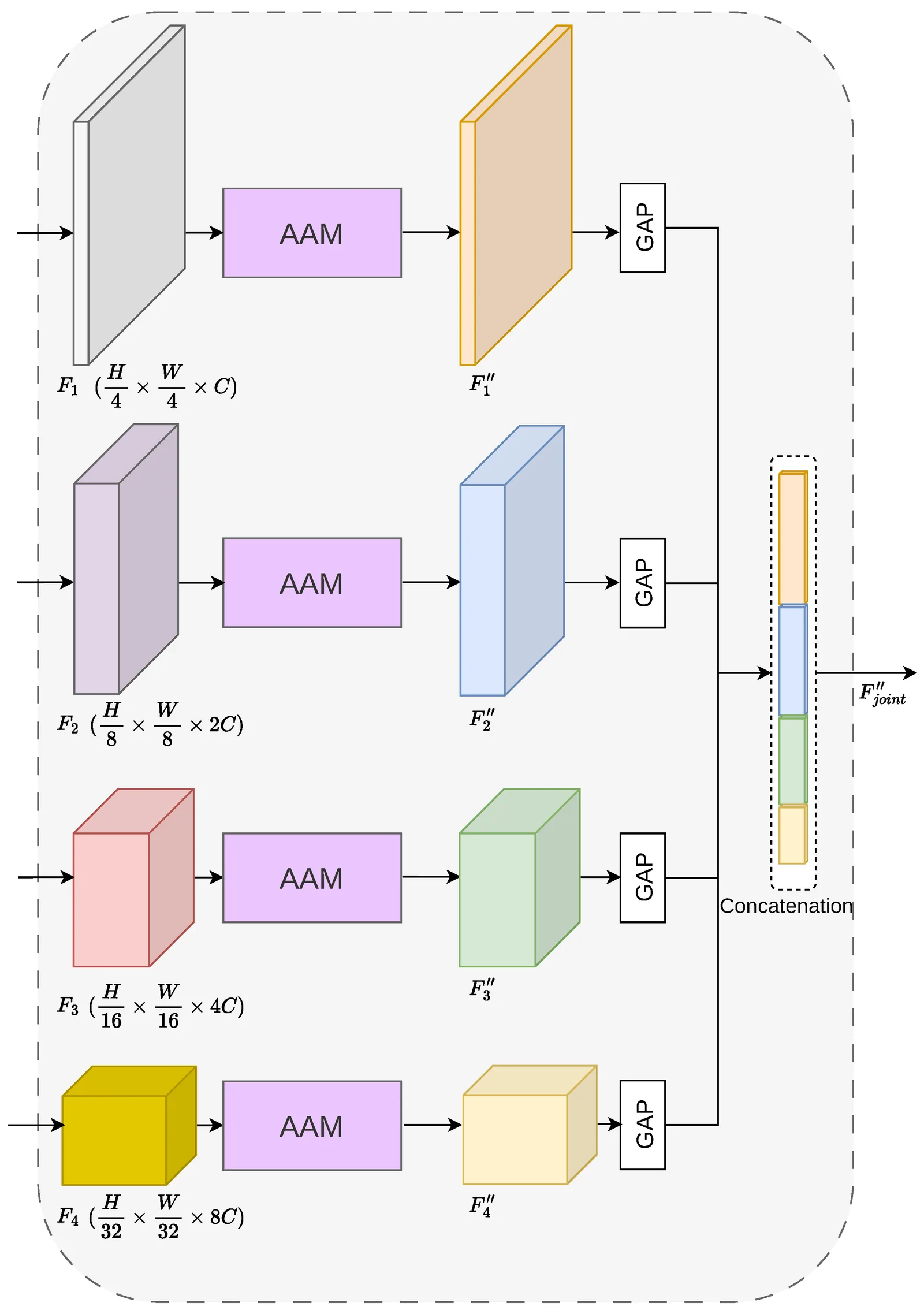
Proposed multiscale attention fusion module.

The proposed MAF module is depicted in Figure 5, highlighting its structural composition. This fusion block incorporates multiple AAM modules. By leveraging the hierarchical architecture of the swin transformer backbone, features extracted from various layers are utilized to generate multiscale feature maps. Initially, these extracted features, which vary in spatial and channel dimensions, are fed into their respective AAM modules to enhance the global feature representation capability of the network.

**Figure 6:**
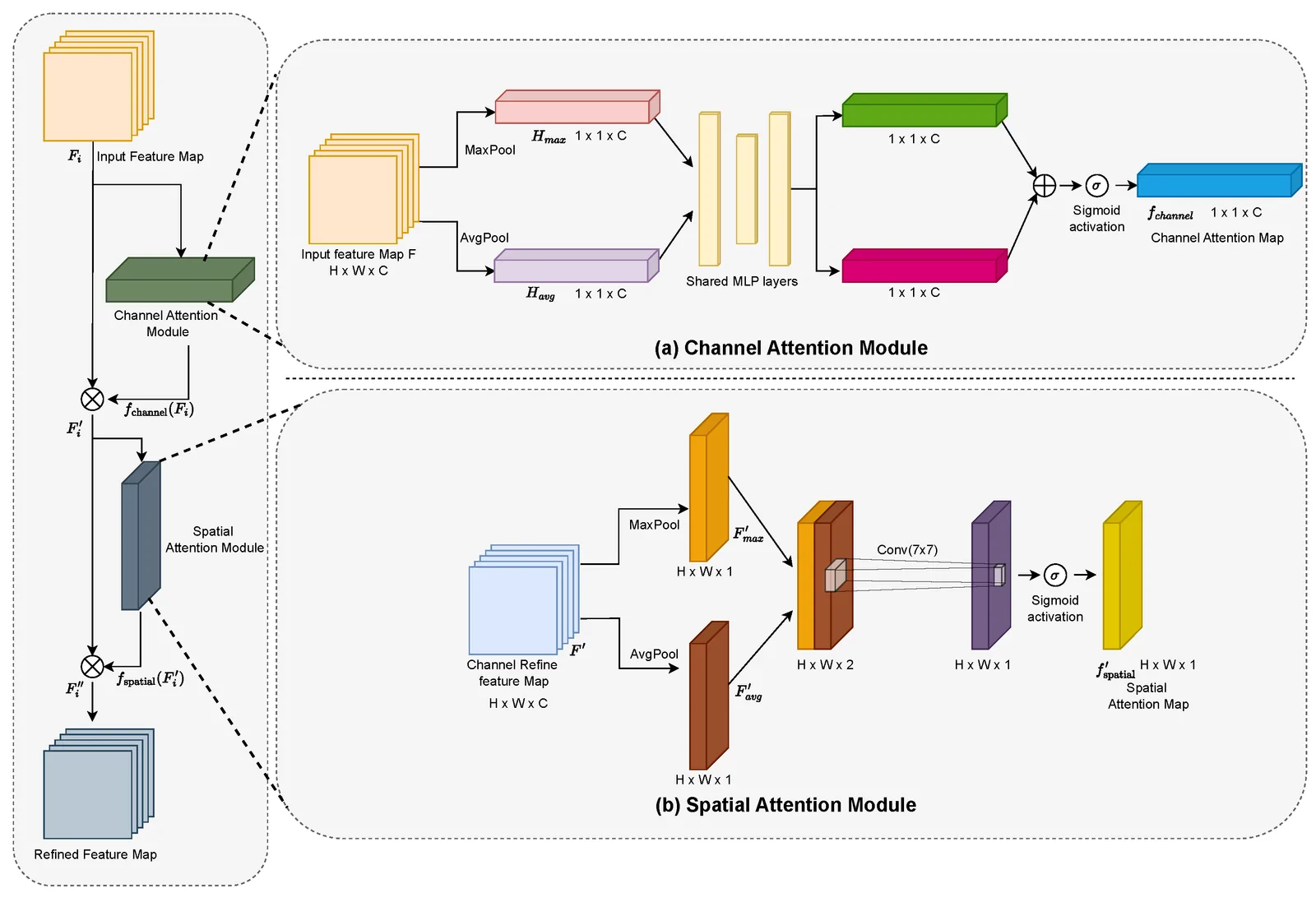
Detailed structure of the adaptive attention module on the left side, comprising two attention mechanisms on the right side: (**a**) channel attention mechanism and (**b**) spatial attention mechanism.

The proposed AAM module comprises channel attention and spatial attention mechanisms, as depicted in Figure 6 The channel attention mechanism distributes its values across the spatial dimensions, enabling spatial feature refinement, while the spatial attention mechanism propagates its values along the channel dimension, enhancing channel-specific feature representation. The MAF module begins with the intermediate feature map $F_{i}$, extracted from the respective output layer of the swin transformer. This feature map is initially processed by the channel attention module, resulting in the refined feature map $F_{i}^{\prime }$. Subsequently, $F_{i}^{\prime }$ is passed to the spatial attention module, which further enhances the representation and produces the final feature map $F_{i}^{\prime \prime }$. It can be mathematically formulated as follows:

(11) $$F_{i}^{\prime }=f_{\mathrm{channel}}\left(F_{i}\right)⊗F_{i}$$

(12) $$F_{i}^{\prime \prime }=f_{\mathrm{spatial}}\left(F_{i}^{\prime }\right)⊗F_{i}^{\prime },$$

where, $f_{\mathrm{channel}}\left(\right)$ and $f_{\mathrm{spatial}}\left(\right)$ represents the channel and spatial attention mechanisms, respectively, and ⊗ denotes element-wise multiplication. Consider a specific example with an input $F_{1}\in R^{\frac{H}{4}\times \frac{W}{4}\times C}$, the channel attention mechanism is represented as a 1D mapping $f_{\mathrm{channel}}\left(F_{1}\right)\in R^{1\times 1\times C}$, while the spatial attention mechanism is a 2D mapping $f_{\mathrm{spatial}}\left(F_{1}^{\prime }\right)\in R^{\frac{H}{4}\times \frac{W}{4}\times 1}$.

In the first step, the input feature map passes through the channel attention module, where it undergoes both average pooling and max pooling operations. Then, the resultant feature maps $H_{avg}\left(F_{1}\right)$ and $H_{max}\left(F_{1}\right)$ are then passed through respective MLP layers. The ReLU activation function is employed after the pooling operation. The outputs from the two pooling operations are subsequently combined using element-wise addition.

In the first step, the input feature map passes through the channel attention module, where it undergoes both average pooling and max pooling operations. Then, the resultant feature maps $H_{avg}\left(F_{1}\right)$ and $H_{max}\left(F_{1}\right)$ are passed through respective shared MLP layers. A ReLU activation function is applied after the first fully connected layer of each MLP. The outputs from the two MLP branches are subsequently combined using element-wise addition.

(13) $$H_{avg}\left(F_{1}\right)=\mathrm{AvgPool}\left(F_{1}\right)$$

(14) $$H_{max}\left(F_{1}\right)=\mathrm{MaxPool}\left(F_{1}\right)$$

(15) $$f_{channel}\left(F_{1}\right)=\sigma \left(MLP\left(H_{avg}\left(F_{1}\right)\right)+MLP\left(H_{max}\left(F_{1}\right)\right)\right)$$

where, σ is the sigmoid function.

(16) $$F_{1}^{\prime }=f_{channel}\left(F_{1}\right)⊗F_{1}$$

To further obtain a refined feature map, $F_{1}^{\prime }$ is passed through the spatial attention mechanism. Where average and max pooling is applied along the channel axis to get $F_{avg}^{\prime }\left(F_{1}^{\prime }\right)$ and $F_{max}^{\prime }\left(F_{1}^{\prime }\right)$, respectively. These feature maps are then concatenated and processed by a 7×7 convolution to obtain the final spatial attention feature map, which can be given by:

(17) $$f_{\mathrm{spatial}}^{\prime }\left(F_{1}^{\prime }\right)=\sigma \left(f_{7\times 7}^{\prime }\left(\mathrm{concat}\left[F_{avg}^{\prime }\left(F_{1}^{\prime }\right),F_{max}^{\prime }\left(F_{1}^{\prime }\right)\right]\right)\right)$$

where, concat denotes channel-wise concatenation and σ represents the sigmoid function. $f_{7\times 7}$ is a convolutional layer with a kernel size of 7×7. Finally, refined feature map $F_{1}^{\prime \prime }$ can be obtained by:

(18) $$F_{1}^{\prime \prime }=f_{\mathrm{spatial}}^{\prime }\left(F_{1}^{\prime }\right)⊗F_{1}^{\prime }$$

Upon passing through the respective AAM module, multiscale feature maps $F_{1}^{\prime \prime },F_{2}^{\prime \prime },F_{3}^{\prime \prime },F_{4}^{\prime \prime }$ with the size of $F_{1}^{\prime \prime }\in R^{\frac{H}{4}\times \frac{W}{4}\times C}$, $F_{2}^{\prime \prime }\in R^{\frac{H}{8}\times \frac{W}{8}\times 2C}$, $F_{3}^{\prime \prime }\in R^{\frac{H}{16}\times \frac{W}{16}\times 4C}$, $F_{4}^{\prime \prime }\in R^{\frac{H}{32}\times \frac{W}{32}\times 8C}$ are extracted. These features are subsequently passed through a global average pooling operation and concatenated along the channel dimension to generate the final feature map $F_{joint}^{\prime \prime }$. The corresponding process is outlined as follows:

(19) $$F_{joint}^{\prime \prime }=\mathrm{concat}\left[\mathrm{AvgPool}\left(F_{1}^{\prime \prime }\right);\mathrm{AvgPool}\left(F_{2}^{\prime \prime }\right);\mathrm{AvgPool}\left(F_{3}^{\prime \prime }\right);\mathrm{AvgPool}\left(F_{4}^{\prime \prime }\right)\right]$$

The joint feature map $F_{\mathrm{joint}}^{\prime \prime }$ is processed by a three-layer MLP classifier, which produces the final classification output. The first two layers consist of 64 neurons each and employ the ReLU activation function to introduce non-linearity. The number of neurons configured in the final layer matches the number of output classes and uses a softmax function.

## 3. Results and Discussions

This section first presents a detailed description of the datasets, followed by the experimental setup and model configurations employed in this simulation study. Subsequently, a comparative analysis is conducted across baseline architectures, including transformer-based and hybrid transformer-based models. Finally, the performance of the proposed model is evaluated against state-of-the-art approaches to establish its efficacy.

### 3.1. Datasets

In this paper, the performance of the proposed HM-Swin model is evaluated using two widely recognized datasets, namely PhysioNet2016 [40] and PhysioNet2022 [41], both of which provide PCG recordings of heart sounds for analysis. PhysioNet2016 database contains 3153 heart sound recordings. Among these, 2488 were labelled as normal, while 665 were categorized as abnormal. The recordings were collected by various teams using different types of electronic stethoscopes in both clinical and non-clinical environments. Due to the uncontrolled nature of the recording settings, the duration of the recordings varied between 5 and 120 s. All recordings were resampled to 2000 Hz. The dataset covers a diverse population, including children, adults, and elderly individuals. Most of the abnormal recordings correspond to coronary and valvular diseases, although other cardiac conditions are also represented. The PhysioNet2022 dataset includes heart sound recordings ranging from 5 to 45 s in length and is accompanied by demographic information such as age group, gender, height, weight, and pregnancy status. It consists of data from 1568 patients, and each patient has up to six heart sound recordings, resulting in a total of 5272 recordings in the full dataset. The recordings were collected from specific auscultation points, including the pulmonary valve, aortic valve, mitral valve, tricuspid valve, and other locations. Each patient is labelled with a heart murmur classification, categorized as present, unknown, or absent.

### 3.2. Experimental Setup and Implementation Details

The proposed HM-Swin network is configured with a linear embedding dimension of 32 channels. The architecture consists of four stages with depths given by M={2,2,6,2}, and the corresponding numbers of self-attention heads are set to {3,6,12,24}. For an input feature map of dimensions 64×64×3, the model employs a patch size of 4×4, partitioning the image into 16×16 non-overlapping patches. Each patch, comprising 48 features (flattened from 4×4×3), undergoes linear projection, yielding a 32-dimensional embedding. The swin transformer-based deep feature extractor blocks process these embeddings, with stage 1 preserving the dimensions of 16×16×32. Subsequent patch merging operations perform hierarchical downsampling, reducing the spatial resolution while doubling the channel dimensions, producing feature maps of sizes 8×8×64, 4×4×128, and 2×2×256 at stages 2, 3, and 4, respectively. The classification head consists of three fully connected layers. The first two layers are fully connected layers, each with 64 neurons and a ReLU activation function. The output layer contains neurons corresponding to the number of target classes and employs the softmax activation function to generate class probabilities. The training and evaluation of the proposed network were conducted on a hardware platform comprising an NVIDIA GeForce RTX 3090. The proposed HM-Swin architecture is implemented using the open-source machine learning TensorFlow library in Python 3. The optimization process employs the Adam optimizer with a learning rate of $10^{-5}$, and the cross-entropy loss function is adopted as the objective function. The network is trained for up to 400 epochs using an early stopping strategy. If validation performance does not improve for 15 consecutive epochs, training stops, and the best weights from the epoch with the highest validation performance are restored. A batch size of 32 is used. The dataset is divided into training, validation, and testing subsets. A 10-fold cross-validation procedure is applied exclusively to the 90% portion allocated for training and validation, whereas the remaining 10% is held out as an independent test set and used only for the final performance evaluation. Additionally, the splitting is carried out on a subject-wise basis to ensure that no subject appeared in more than one subset within the same fold, thereby eliminating any chance of data leakage. The proposed HM-Swin model generates one prediction for each heart sound recording. Specifically, for the PhysioNet2022 dataset, multiple recordings from the same patient are evaluated separately and are not aggregated into a single patient-level prediction. Therefore, the reported results represent recording-level performance under the adopted subject-wise evaluation protocol and should not be interpreted as patient-level performance.

### 3.3. Comparison with Transformer-Based Methods and Hybrid Transformer Model

The primary objective of this subsection is to evaluate the performance of the various transformer-based models in heart sound classification. This study employs variants of ViT and swin transformer models to perform classification on heart sound datasets, namely PhysioNet2016 and PhysioNet2022. The model’s performance has been rigorously evaluated using various metrics, including accuracy, F1-score, sensitivity, specificity, and an overall classification score. These performance metrics are calculated from the confusion matrix using the conventional definitions based on true-positive, true-negative, false-positive, and false-negative predictions. The score is computed as the arithmetic mean of sensitivity and specificity, given by score=(sensitivity+specificity)/2. For PhysioNet2022, the F1-score is reported as the macro average across the three classes.

Table 1 compares the models using the PhysioNet2016 dataset, while Table 2 presents the results based on the PhysioNet2022 dataset. Mainly 4 transformer based models are considered for the performance comparision namely, ViT-Base, ViT-Large, Swin-Base, and Swin-Large. Additionally, we also explored a hybrid model, Inception-v3 + ViT, where the output features extracted by Inception-v3 are further processed through the ViT network. As the PhysioNet2022 dataset comprises three distinct heart sound categories, we opted for weighted accuracy to evaluate the model’s performance. The following provides a comprehensive overview of the models utilized in this study:

- *ViT-Base:* The ViT-Base architecture utilizes a patch size of 16×16 pixels with an embedding dimension of 768. It employs 12 attention heads and an MLP with the dimension of 3072. The model has a depth of 12 transformer layers, which refers to the total number of layers in the transformer encoder stack.
- *ViT-Large:* The ViT-Large variant maintains the same patch size of 16×16 but increases the embedding dimension to 1024. It expands the number of attention heads to 16, incorporates a larger MLP dimension of 4096, and features a deeper architecture with 24 transformer layers.
- *Swin-Base:* The Swin-Base model adopts a patch size of 4×4, with an initial embedding dimension of 128. It consists of 12 attention heads, and the size of the MLP is 512. The network is structured with a depth configuration of [2, 2, 18, 2] across its hierarchical stages.
- *Swin-Large:* The Swin-Large model follows the same patch size of 4×4 but increases the embedding dimension to 192. It employs 16 attention heads, and has an MLP dimension expanded to a size of 768, compared to Swin-Base. The architecture consists of [2, 2, 36, 2] transformer layers.
- *Inception-v3 + ViT:* This model adopts a hybrid approach by integrating Inception-v3 as a feature extractor, followed by a ViT encoder. The Inception-v3 network process the input images of size 224×224×3. The extracted feature maps are then transformed into patch embeddings using a 1×1 convolutional layer, reducing the feature dimension to 512. The ViT encoder has 12 transformer layers, each containing 8 attention heads in the MSA.

Table 1 and Table 2 demonstrate that ViT-Base yields the lowest classification performance among the all evaluated models, whereas ViT-Large achieves a marginal improvement in overall performance over the ViT-Base model. Specifically, ViT-Large improves the classification accuracy by 1.39 and 0.07 compared to the ViT-Base model for Physionet2016 and Physionet2022 datasets, respectively. For the swin transformer variants, Swin-Base and Swin-Large attain accuracies of 94.78% and 95.43%, respectively, on the PhysioNet2016 dataset, and 84.70% and 84.95% on the PhysioNet2022 dataset. Across both the datasets, swin models consistently outperform ViT models, demonstrating their superior capability in heart sound classification. The hybrid model, Inception-v3 + ViT, further enhances performance, achieving improvements of 3.57, 2.18, 1.15, and 0.5 over ViT-Base, ViT-Large, Swin-Base, and Swin-Large on the PhysioNet2016 dataset. A similar trend is observed for the PhysioNet2022 dataset. This performance enhancement is attributed to the hybrid architecture, where Inception-v3, as a CNN-based feature extractor, efficiently captures local spatial patterns, while ViT refines these features through its global self-attention mechanism. However, despite its improved classification accuracy, this cascaded hybrid approach increases the parameter count of the model, leading to higher computational complexity.

The effectiveness of the proposed HM-Swin model is substantiated by its superior performance over existing architectures, including ViT-Base, ViT-Large, Swin-Base, Swin-Large, and the hybrid Inception-v3+ViT model. Specifically, on the PhysioNet2016 dataset, the HM-Swin model achieves accuracy enhancements of 5.56, 4.57, 3.54, 2.89, and 2.39, respectively, when compared to these models. Likewise, for the PhysioNet2022 dataset, the HM-Swin model consistently surpasses ViT-Base, ViT-Large, Swin-Base, Swin-Large, and hybrid Inception-v3+ViT, yielding weighted accuracy improvements of 7.58, 7.51, 5.86, 5.61, and 4.47, respectively. These results demonstrate the efficacy of the hierarchical multiscale swin transformer with attention fusion in enhancing heart sound classification accuracy. In addition, unlike hybrid ViTs, which often rely on external architectures like CNNs for local feature extraction, the multiscale swin transformer inherently incorporates spatial hierarchies and multi-level attention within a single unified framework, leading to improved accuracy and computational efficiency.

**Figure 7:**
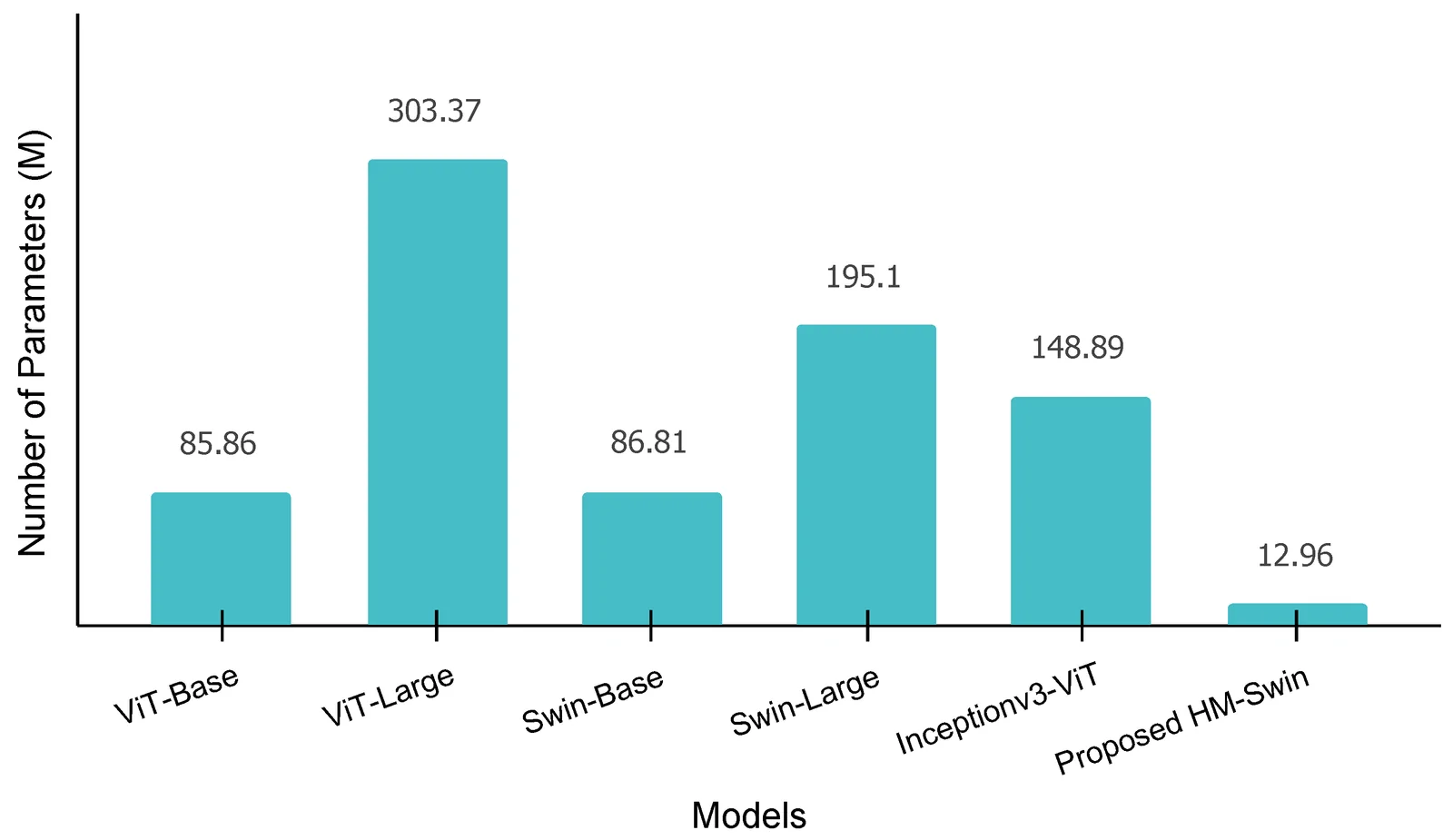
Comparison of training parameters (in millions) between transformer-based, hybrid models, and the proposed HM-Swin.

Figure 7 provides a comparative analysis of the training parameters across different models. The ViT-Base and ViT-Large models require 85.86 million and 303.37 million parameters, respectively. Although ViT-Large demonstrates a marginal improvement over ViT-Base, this comes at the expense of a significantly higher parameter count, thereby emphasizing the tradeoff between model complexity and computational efficiency. The Swin-Base model, with 86.81 million parameters, exhibits computational requirements comparable to ViT-Base, whereas the Swin-Large model, requiring 195.10 million parameters, remains significantly more efficient than ViT-Large. Furthermore, the hybrid Inception-v3 + ViT model requires 148.89 million parameters. In contrast, the proposed HM-Swin model exhibits remarkable efficiency, requiring only 12.96 million parameters while achieving significantly higher performance. The model is designed to be both lightweight and highly accurate, which is suitable for medical applications. The feature maps from all four stages of the swin backbone are retained to preserve hierarchical representations at different spatial resolutions and are subsequently processed by the multiscale attention fusion module. In this study, HM-Swin is evaluated as an integrated architecture rather than through separate component-wise analysis. Therefore, in the absence of ablation experiments, the individual contributions of the attention branches cannot be determined.

### 3.4. Comparison with State-of-the-Art Methods

Table 3 and Table 4 present the classification accuracy and the number of parameters of the proposed model in comparison with state-of-the-art methods on the PhysioNet2016 and PhysioNet2022 datasets, respectively. More specifically, to assess the effectiveness of the proposed multiscale fusion architecture, various transformer-based and hybrid transformer-based models are considered. The tables present results from independently published studies using the same publicly available datasets. These studies may differ in their input feature representations, dataset partitions, pretraining strategies, and other experimental settings, as detailed in the respective cited publications.

As summarized in Table 3, the proposed hierarchical HM-Swin achieves a notable accuracy of 98.32%, representing an improvement of 7.32% over Swin-T, which achieves 91.0%, and an enhancement of 5.32% and 4.92% over PCTMF-Net (93.0%) and DCv-Swin (93.4%), respectively. This significant gain highlights the capability of the proposed model to effectively leverage multiscale features for heart sound classification. In terms of model efficiency, HM-Swin also demonstrates superiority by utilizing only around 13M parameters, which is approximately half of the parameter count of DCv-Swin (26.71M) and Swin-T (26.65M). Furthermore, it requires fewer parameters than PCTMF-Net (18.95M), despite achieving considerably higher accuracy. Remarkably, the proposed model outperforms the second-best approach, DCv-Swin, by a margin of 5.8%, while using less than half the number of parameters. The superior performance of the proposed HM-Swin model can be attributed to its multiscale feature extraction capabilities, which effectively leverage MFCC and delta features in conjunction with attention modules. Unlike DCv-Swin and other transformer-based architectures, the hierarchical design of HM-Swin effectively captures diverse feature representations at different scales. This multiscale fusion strategy ensures both high classification accuracy and computational efficiency, making it a compelling choice for heart sound analysis.

As presented in Table 4, the ViT model, utilizing a spectrogram-based feature representation, exhibits the lowest classification performance, highlighting the limitations of standalone transformer-based approaches. The results highlight the significant improvement achieved by the HM-Swin model, which delivers an accuracy of 90.56%, representing a 21.56 increase compared to the ViT model [43], which achieves 69% accuracy. In contrast, the hybrid transformer approaches, such as the parallel-attentive model [33] and the dual-transformer model [44], achieve higher accuracies of 79.8% and 83.2%, respectively. These models benefit from integrating CNNs or leveraging pre-trained audio representations, but their performance comes at the cost of significantly increased model complexity, requiring approximately 27.81M and 172.7M parameters, respectively. Notably, the dual-transformer model employs late fusion techniques with pre-trained models, namely, audio spectrogram transformer (AST) and masked modeling duo (M2D), which enhances accuracy but results in a 13-fold increase in parameter count compared to the HM-Swin model. The proposed HM-Swin model surpasses the accuracy of both standalone transformer and hybrid transformer architectures while maintaining a notably lower parameter count of only around 13M. This efficiency is attributed to its multiscale transformer architecture, which effectively captures inter-modality representations and hierarchical features, addressing the limitations of existing hybrid approaches. In comparison, the parallel-attentive model achieves an accuracy of 79.8% but requires more than twice the parameters of HM-Swin. Similarly, the dual-transformer model, while attaining an accuracy of 83.2%, lacks the capability to exploit multiscale features due to its reliance on late fusion of pre-trained models. In summary, the HM-Swin model demonstrates superior performance and efficiency for heart sound classification on the PhysioNet2022 dataset. Its ability to achieve competitive accuracy with substantially fewer parameters underscores its potential for resource-constrained applications.

## 4. Conclusions

In this study, we proposed HM-Swin, an attention-aided hierarchical multiscale swin transformer network for heart sound classification. We also designed a multiscale attention fusion block that contains multiple AAM (adaptive attention module) to effectively integrate diverse feature representations. By leveraging the swin transformer as a backbone, the model captures hierarchical and multiscale features, while the attention fusion module effectively integrates contextual information across multiple scales. The AAM, with its channel-wise and spatial attention mechanisms, further refines these features, ensuring a robust classification performance. Extensive experimental results demonstrate that the proposed model achieves a superior classification performance compared to other baseline transformer and hybrid models. The proposed HM-Swin model consistently outperforms existing state-of-the-art methods, in terms of classification accuracy on the PhysioNet2016 and PhysioNet2022 datasets. Furthermore, the proposed framework, despite its promising performance, maintains architectural compactness, as evidenced by its reduced number of training parameters.

## Figures and Tables

**Table 1:** Performance comparison of transformer-based methods and hybrid transformer models for heart sound classification on the PhysioNet2016 dataset.

Category	Method	Accuracy	F1 Score	Score	Sensitivity	Specificity
Transformer-Based Methods	ViT-Base	92.36	95.04	88.38	95.98	80.78
	ViT-Large	93.75	95.92	91.04	96.22	85.86
	Swin-Base	94.78	96.62	91.79	97.50	86.08
	Swin-Large	95.43	97.03	92.54	98.05	87.03
Hybrid Methods	Inception-3 + ViT	95.93	97.19	95.86	95.99	95.73
Proposed	HM-Swin	**98.32**	**98.89**	**97.89**	**98.71**	**97.07**

**Table 2:** Performance comparison of transformer-based methods and hybrid transformer models for heart sound classification on the PhysioNet2022 dataset.

Category	Method	Weighted Accuracy	F1 Score	Score	Sensitivity	Specificity
Transformer-Based Methods	ViT-Base	82.98	55.34	65.19	52.22	78.16
	ViT-Large	83.05	58.51	67.49	54.70	80.28
	Swin-Base	84.70	63.25	71.20	59.43	82.96
	Swin-Large	84.95	65.23	72.54	61.45	83.63
Hybrid Methods	Inception-v3 + ViT	86.09	73.68	81.28	73.92	88.63
Proposed	HM-Swin	**90.56**	**79.81**	**83.74**	**78.12**	**89.35**

**Table 3:** Performance comparison of state-of-the-art approaches on the PhysioNet2016 dataset.

Method	Feature	Accuracy	Number of Parameters
Swin-T [42]	mel-spectrogram	91.0	26.65M
PCTMF-Net [36]	second-orders spectral features	93.0	18.95M
DCv-Swin [42]	mel-spectrogram	93.4	26.71M
Proposed HM-Swin	MFCCs, delta and double delta	**98.32**	**12.96M**

**Table 4:** Performance comparison of state-of-the-art approaches on the PhysioNet2022 dataset.

Method	Feature	Weighted Accuracy	Number of Parameters
ViT [43]	Spectrogram	69	85.52M
Parallel-attentive model [33]	Mel spectrogram	79.8	27.81M
Dual-Transformer [44]	Log-mel spectrogram	83.2	172.7M
Proposed HM-Swin	MFCCs, delta and double delta	**90.56**	**12.96M**

## Data Availability

The data used in this study are publicly available from the respective websites: https://physionet.org/content/challenge-2016/1.0.0/ (accessed on 29 September 2026), and https://moody-challenge.physionet.org/2022/ (accessed on 29 September 2026).
